# Transcriptomics reveal an integrative role for maternal thyroid hormones during zebrafish embryogenesis

**DOI:** 10.1038/s41598-017-16951-9

**Published:** 2017-11-30

**Authors:** Nadia Silva, Bruno Louro, Marlene Trindade, Deborah M. Power, Marco A. Campinho

**Affiliations:** Comparative Endocrinology and Integrative Biology Group, Centre for Marine Sciences (CCMAR), Universidade do Algarve, Faro, Portugal

## Abstract

Thyroid hormones (THs) are essential for embryonic brain development but the genetic mechanisms involved in the action of maternal THs (MTHs) are still largely unknown. As the basis for understanding the underlying genetic mechanisms of MTHs regulation we used an established zebrafish monocarboxylic acid transporter 8 (MCT8) knock-down model and characterised the transcriptome in 25hpf zebrafish embryos. Subsequent mapping of differentially expressed genes using Reactome pathway analysis together with *in situ* expression analysis and immunohistochemistry revealed the genetic networks and cells under MTHs regulation during zebrafish embryogenesis. We found 4,343 differentially expressed genes and the Reactome pathway analysis revealed that TH is involved in 1681 of these pathways. MTHs regulated the expression of core developmental pathways, such as NOTCH and WNT in a cell specific context. The cellular distribution of neural MTH-target genes demonstrated their cell specific action on neural stem cells and differentiated neuron classes. Taken together our data show that MTHs have a role in zebrafish neurogenesis and suggest they may be involved in cross talk between key pathways in neural development. Given that the observed MCT8 zebrafish knockdown phenotype resembles the symptoms in human patients with Allan-Herndon-Dudley syndrome our data open a window into understanding the genetics of this human congenital condition.

## Introduction

In tetrapods thyroid hormones (THs) have a key role in the development of the brain^1–3^ and in rodents multiple components of the thyroid cellular signalling pathway and T3-induced gene expression^4,5^ occur^6^. Collectively the evidence indicates that TH signalling in developing rodent brain cells acts when T3 binds to thyroid hormone nuclear receptors and transactivate or represses gene expression. However, the role of THs, especially maternal THs (MTHs), in vertebrate embryonic development, and notably neural development, is still largely unexplored^7^.

MTHs act before a functional thyroid gland develops. In mammals the placenta tightly regulates TH availability to the foetus^8^. Evidence from several teleost species indicate that MTHs are deposited in eggs and that^9–11^ thyroid hormone receptors (TRs) are expressed during early embryonic development^10,12–15^. Studies in zebrafish seem to suggest that TRs repress retinoic acid signalling in a T3-independent manner during early development^12,13^. Furthermore, inappropriate levels of thyroid hormone during embryonic development negatively influence neurogenesis, myelination, dendrite proliferation and synapse formation (reviewed in^16,17^).

The importance of the TH-specific membrane transporter MCT8 (SLC16A2) for neurodevelopment in humans was revealed by the severe global neurological impairments identified in subjects with X-linked Allan-Herndon-Dudley syndrome (AHDS). MCT8 is present in zebrafish embryos in the developing nervous system and has a high affinity for the active form of the THs, T3^18^. In three independent studies conditional knockdown of the MCT8 transporter with morpholinos during zebrafish development caused severe disruption of brain development similar to AHDS^15,19,20^, an outcome that has not been achieved in any murine model so far^21–23^. Although, the zebrafish mutant for MCT8 does not replicate the human phenotype of AHDS^24^. The evidence from the zebrafish MCT8 knockdown model indicates that maternal THs in teleost eggs have an essential role in neural development. Our previous studies revealed that the zebrafish MCT8 knockdown model display locomotor, cellular and molecular changes congruent with the consequences of AHDS in humans^15,21,25^.

Here we unmask MTH-dependent developmental mechanisms using morpholino-mediated knockdown of MCT8 to prevent MTH from entering target cells in developing zebrafish embryos^15^. The transcriptome of developing control and MCT8 morphants in 25 hours post-fertilization (hpf) zebrafish embryos was established and identified gene networks under the regulation of MTHs during zebrafish development. Differentially expressed neural genes were mapped *in situ* in embryos and cells under the regulation of MTHs during zebrafish development were identified.

## Results

### MTHs regulate a wide variety of genes and gene networks during zebrafish embryogenesis

**Figure 1:**
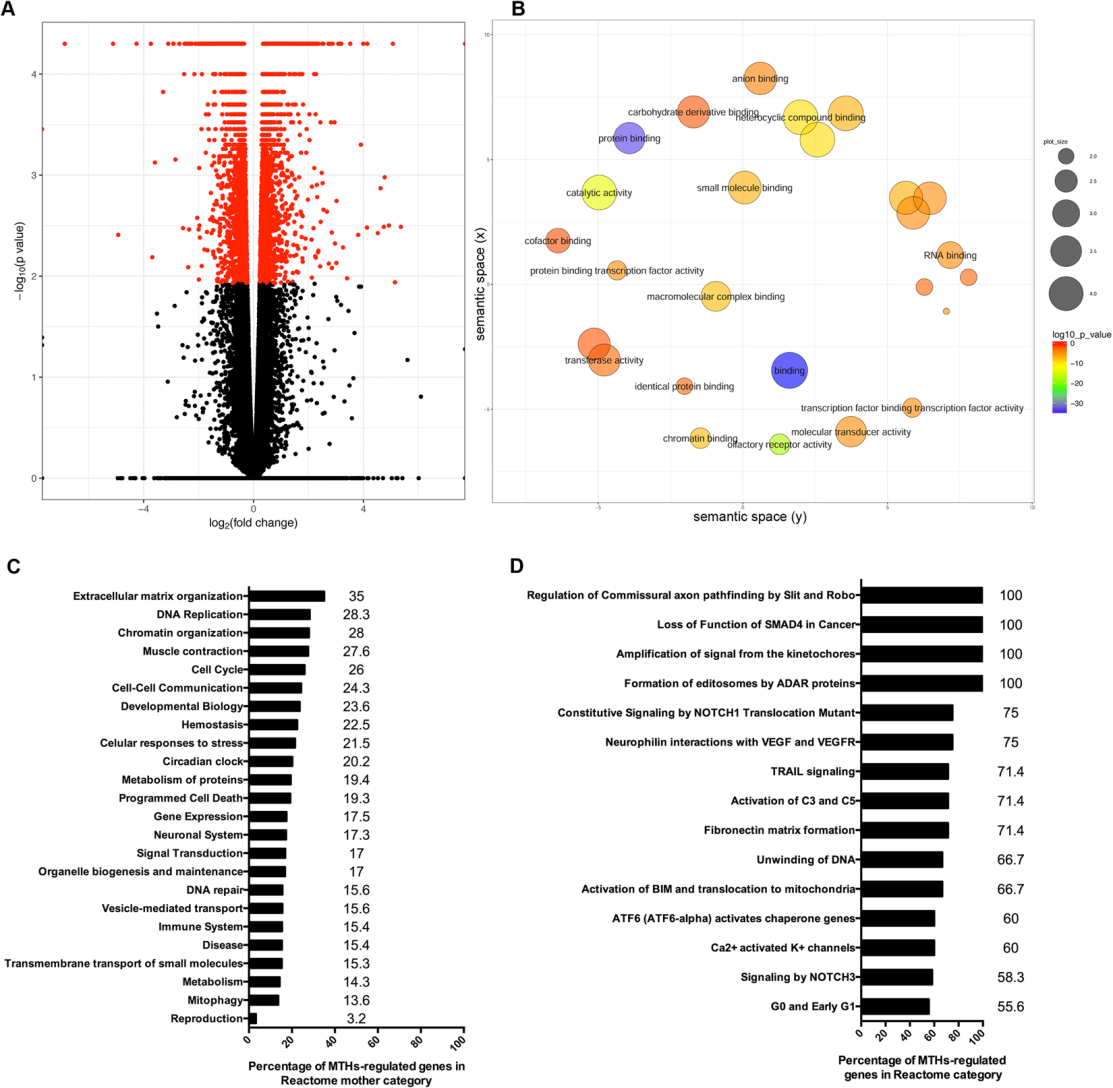
Zebrafish MTH-dependent transcriptome and pathway analysis at 25hpf. (**A**) Volcano plot and (**B**) molecular function GO enrichment analysis slimmed with semantic clustered with REVIGO of differentially expressed genes between 25hpf zebrafish embryos microinjected with either control or MCT8 MO. In (**A**) red points represent significantly expressed genes (p < 0.01; FDR < 0.05). In (**B**) circle size represents the frequency of GO terms and the colour scale represents log10 P-value. At 25hpf MTHs regulated the expression of genes in all Reactome pathways during zebrafish embryogenesis. (**C**) Graphical representation of the ratio of MTH-regulated genes in the mother pathway at 25hpf of zebrafish embryogenesis. (**D**) Graphical representation of the Reactome pathways most populated by MTH-regulated genes. The number near the bars in (**C**) and (**D**) denote the exact numeric value depicted by the bar in the graphs.

On average more than 21 million pair end sequences were produced per sample and overall 2.2% of the sequences were removed after quality control editing. Comparisons between the MCT8 morpholino (MO) and control (CTR) samples of 85,652 paired assembled transcripts (Fig. 1A and Supplementary Fig. 1A): 1) generated 3,462 transcripts with a differential FPKM; 2) 41,475 of the assembled transcripts represented coding sequences and 2,921 had a differential FPKM; 3) out of 35,217 annotated genes, 4,876 had a differential FPKM (FDR < 0.05, p < 0.01) and 38 arose from differential promoter usage, and; 4) from 53,350 primary transcripts sharing transcription start sites, 4,343 had a differential FPKM. Most of the differentially expressed genes between control and MCT8 morphants were less than 2-fold different albeit with high statistical significance (Fig. 1A and Supplementary Fig. 1A; p < 0.01; FDR < 0.05).

Differentially expressed genes between the control and MCT8 morphants at 25hpf were assigned to 27 statistically significant (p < 0.05; allowed medium semantic similarity) non-redundant molecular function GO-term categories (Fig. 1B and Supplementary Table 1). Overall, binding was the most populated category followed by transcription factor activity and catalytic activity (Fig. 1B and Supplementary Table 1). In the biological process category, the subordinate categories, cellular, single organism, metabolic and developmental processes were dominant (Supplementary Fig. 1B and Supplementary Table 1).

Pathway analysis using the Reactome V58 (www.reactome.org)^26^ human curated database, revealed that differentially expressed genes were involved in 1681 pathways of which 15 had more than 55% of their members regulated by MTHs (Fig. 1C). In the Reactome mother category pathways the percentage of regulated genes varied from 35% (Extracellular matrix organization) to 3.2% (Reproduction; Fig. 1C). Notably, in the category, signal transduction, MTHs regulated 17% of all genes and these were present in all subcategories (Fig. 1C). A more focused analysis of each Reactome mother category indicated that MTHs were highly important in regulation of commissural axon guidance by Slit and Robo, SMAD4 regulation, and amplification of signal from the kinetochores, and all genes involved were regulated by MTHs (Fig. 1D, Supplementary Table 2). In the sub-pathway neurophilin interaction with VEGF and VEGFR, 75% of all genes had their expression regulated by MTHs at 25hpf (Fig. 1D and Supplementary Table 2 - R-HSA-194306). Genes belonging to the sub-pathway fibronectin matrix formation (71%, Fig. 1D), unwinding of DNA in replication (66.7%, Fig. 1D) and G0 and early G1 stages of the cell cycle (>55%; Fig. 1D), were also affected by MTH signalling.

Developmental related pathways such as NOTCH, showed a high number of MTH regulated genes, such as NOTCH3 signalling (58.3%; Fig. 1D and Supplementary Table 2 - R-HSA-1980148), NOTCH 1 signalling (32%; Supplementary Fig. 2 and Supplementary Table 2 - R-HSA-1980143) and pre-NOTCH receptor expression and processing (26%; Supplementary Table 2 - R-HSA-1912422). Embryogenesis important WNT and Hedgehog signalling pathways were significantly regulated by MTHs, although fewer genes had significant different expression between control and MCT8 morphants. In the WNT pathway 21% of the genes were differentially expressed in the MTH knockdown (Supplementary Table 2). In the Hedgehog pathway MTH-regulated genes accounted for 23% of the genes in the pathway (Supplementary Table 2). Regulation of Wnt1 biogenesis and trafficking was the most affected WNT sub-pathway and 36% of the genes were differentially expressed between control and MCT8 morphants (Supplementary Fig. 3 and Supplementary Table 2 - R-HSA-3238698). Notably, all steps in the Wnt1 biogenesis and trafficking pathway were affected by MCT8 knockdown (Supplementary Fig. 3). TCF-dependent signalling in response to WNT sub-pathways was also regulated by MTHs (19%; Supplementary Table 2 - R-HSA-201681). However, in this case, MTHs seemed to have a greater effect on TCF nuclear signalling than on any other component of this sub-pathway. The beta-catenin independent WNT signalling sub-pathway was also significantly regulated by MTHs (19%; Supplementary Table 2 - R-HSA-3858494). Although the regulation of genes in this sub-pathway was biased towards genes involved in the cellular apical-basal axis and response to WNT ligands. Degradation of beta-catenin by the destruction complex was the WNT sub-pathway with fewest MTH-regulated genes although almost all reactions of this pathway were affected (16%; Supplementary Table 2 - R-HSA-195253).

In the Hedgehog sub-pathways, MTHs were involved in regulating the expression of 20–22% of the genes (Supplementary Table 2). Notably, the majority of reactions in the Hedgehog sub-pathways contained MTH-regulated genes. Ligand biogenesis was the Hedgehog sub-pathway with the largest number of genes in which expression was regulated by MTHs (22%; Supplementary Table 2 - R-HSA-5358346). MTHs were also involved in regulating genes in Hedgehog off- and on- state sub-pathways (respectively 21 and 20%; Supplementary Table 2 - R-HSA-5610787; R-HSA-5632684).

### MTHs are involved in late neural differentiation, neural diversity and neural progenitor maintenance

We previously reported that MTHs were mainly involved in neural development in zebrafish^15^. In order to identify the cells responsive to MTHs during zebrafish embryogenesis we selected differentially expressed genes (p < 0.01; FDR 5%) involved in zebrafish neural development related to WNT and NOTCH pathways, neural stem cell and neuron differentiation. The selected neural genes (log2[fold-change] values) were: *pax6a* (−0.57), *wnt1* (0.89), *wls* (0.40), *rorab* (-0.54), *her2* (-1.148), *deltaA* (-0,742), *neurod6b* (-1.52) and *gad1b* (-0,591). Whole mount *in situ* hybridization (WISH; *wnt1*, *wls*, *her2*, *pax6a*, *neurod6b*, *rorab*) and whole mount immunohistochemistry (WIHC; DeltaA) of selected genes involved in neural development further confirmed the transcriptome results.

**Figure 2:**
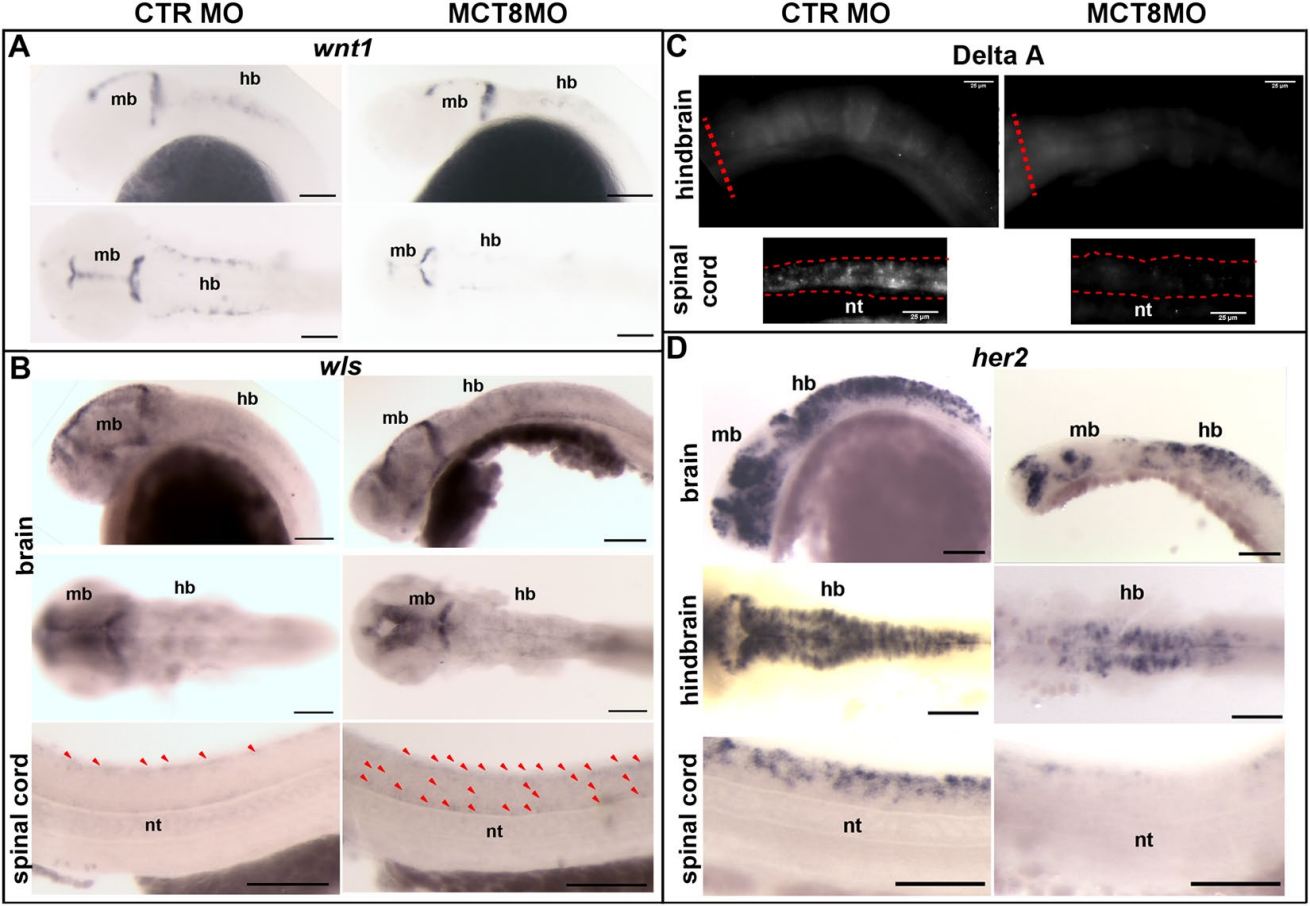
MTHs regulate the WNT and NOTCH pathway genes during zebrafish embryogenesis. (**A**) WISH expression analysis of *wnt1*. Lateral (upper panel) and dorsal images of the brain (lower panel) in control and MCT8 morphant zebrafish embryos at 25hpf. (**B**) WISH expression of the WNT1 protein secretion regulator *- wls*, lateral and dorsal images of the brain and lateral images of the spinal cord in control and MCT8 morphant zebrafish embryos at 25hpf. The red arrows denote *wls* - expressing cells in the spinal cord. (**C**) Fluorescent WIHC expression analysis of the NOTCH ligand DeltaA in the hindbrain and spinal cord of control and MCT8 morphant embryos at 25hpf. (**D**) WISH expression analysis of the NOTCH pathway target gene *her2* in control and MCT8 morphant zebrafish embryos at 25hpf. Lateral and dorsal images of the brain (first and second panels) and lateral images of the spinal cord are shown (lower panel). hb-hindbrain; mb-midbrain; nt-notochord. In (**A**,**B**,**D**) the scale bars represent 100 μm. In (**C)** the scale bars represents 25 μm.

The expression of the WNT ligand and neurogenesis positive regulator *wnt1* ^27^ was increased in the MCT8 morphants. In fact, WISH analysis revealed that at 25hpf in the hindbrain roof plate *wnt1* was downregulated in MCT8 morphants whereas the midbrain expression field expanded (Fig. 2A). In contrast, at 48hpf the expression of *wnt1* increased in the hindbrain roof plate (red arrowheads) and midbrain of MCT8 morphants (Supplementary Fig. 4A).

The gene that regulates the secretion of WNT ligands, *wls*,was also up-regulated^28^, indicating MTHs strongly modulated WNT-signalling. At 25hpf *wls* had a low expression in most brain regions with a slightly higher expression in the second ventricle boundaries of the midbrain (Fig. 2B). In the MCT8 morphants *wls* expression increased in all brain regions but the increase was most evident in the posterior hindbrain ventral floor (Fig. 2B). The expression of *wls* was also up-regulated in the spinal cord (SC) of MCT8 morphants whereas in 25hpf control morphant embryos *wls* expression was only found in dorsal SC cells (Fig. 2B). Expression of *wls* in MCT8 morphants also occurred in intermediate and ventral SC cells (red arrowheads in Fig. 2B). At 48hpf, there was an overall decrease in *wls* expression in MCT8 morphants compared to the control morphant embryos (Supplementary Fig. 4B). Notably, in 48hpf MCT8 morphants lower *wls* expression was most pronounced in the hindbrain and in the SC (Supplementary Fig. 4B). In the SC of control embryos *wls* was expressed in dorsal and ventral cells, a pattern that was maintained in the MCT8 morphants even though the number of *wls*-positive SC cells decreased (Supplementary Fig. 4B). In rescue experiments both *wnt1* and *wls* expression in 25hpf embryos (Supplementary Fig. 7A and B) was similar to the control. These results reinforce the notion that MTHs are involved in the modulation of WNT during neural development in zebrafish.

Reactome analysis of the RNA-seq results revealed that several NOTCH pathway genes were targets of MTHs (Supplementary Figs. 2 and Supplementary Table 2) including the NOTCH ligand *deltaA*. A general decrease in DeltaA immuno-staining was observed in the hindbrain of 25hpf and 48hpf MCT8 morphants (Fig. 2C)^29^. The DeltaA expression decreased most dramatically in the dorsal SC at 25hpf (Fig. 2C and Supplementary Fig. 4C) and by 48hpf this decrease was evident throughout the entire SC (Fig. 2C). Analysis of *her2* expression indicated that the NOTCH pathway was a major target of MTHs during neural development. At 25hpf and 48hpf expression of *her2* was extensively downregulated in both the brain and SC (Fig. 2D and Supplementary Fig. 4D). At 25hpf, the hindbrain was specially affected and the expression field and intensity of *her2* expression was decreased (Fig. 2D). Ventral hindbrain expression of *her2* was almost abolished in MCT8 morphants (Fig. 2D). The *her2* expression was least affected in the forebrain-midbrain boundary of the MCT8 morphants (FMB; Fig. 2D), nonetheless the dorsal FMB domain was more affected than the ventral domain (Fig. 2D). At 48hpf the expression domain of *her2* in MCT8 morphant embryos was totally distinct from control morphant siblings indicating severe changes occurred in brain cellular organization (Supplementary Fig. 4D). In MCT8 morphants the expression of *her2* in the optic tectum was broader but restricted to the midline. In the MCT8 morphants *her2* expression was disorganized in the hindbrain and rather than a single cell layer surrounding the 3rd ventricle, a broader band of cells was evident (Supplementary Fig. 4D). The SC ventral and medial expression domains of *her2* were abolished although some cells in the dorsal domain still expressed *her2* (Supplementary Fig. 4D). In rescue embryos at 25hpf, DeltaA and *her2* were fully rescued to control levels further confirming a role for MTHs in NOTCH signalling during zebrafish neurogenesis (Supplementary Fig. 7C and D).

**Figure 3:**
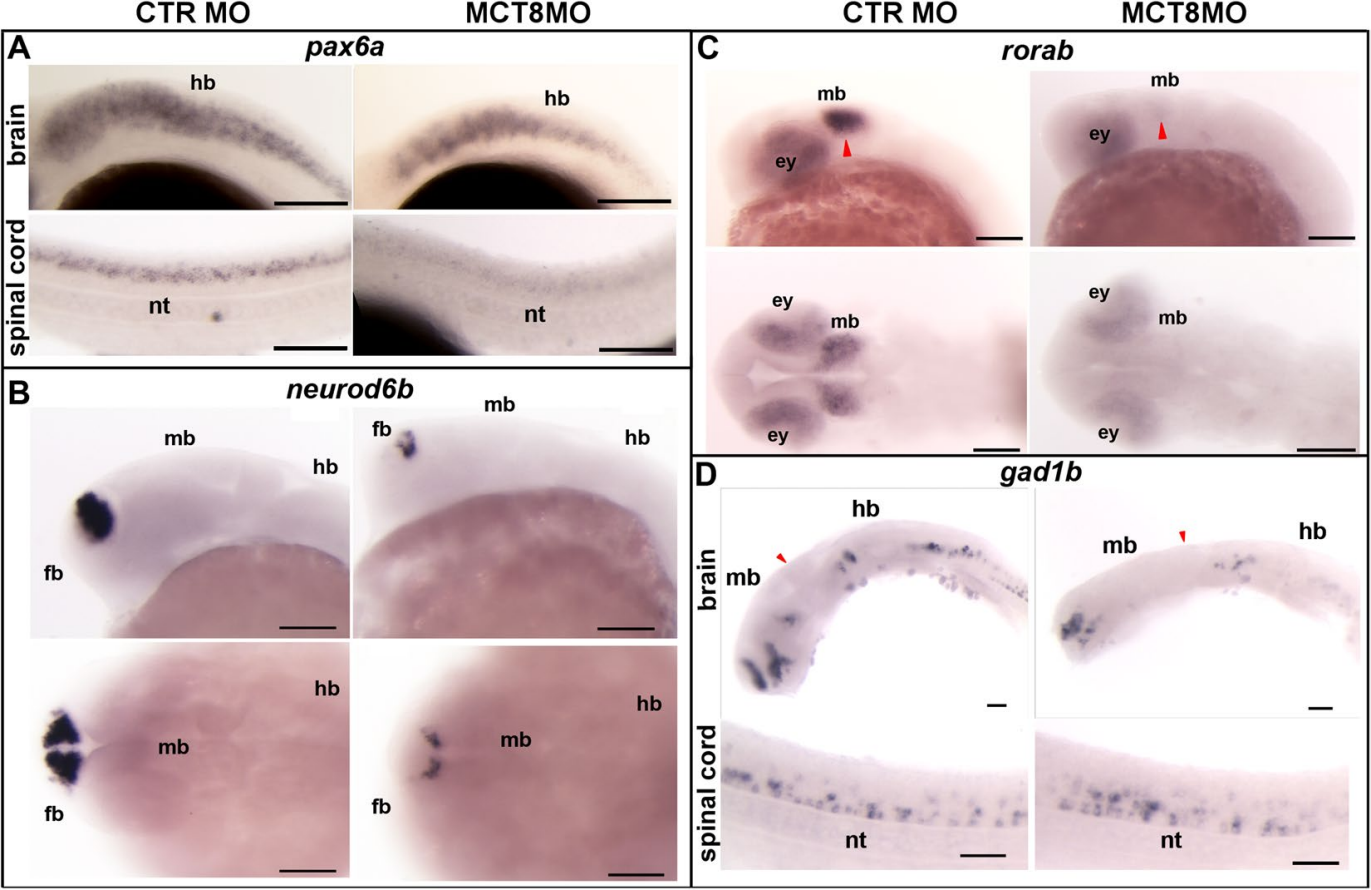
MTHs were involved in zebrafish neural development. The scheme presents a comparison between the control and MCT8 morphant zebrafish embryos at 25hpf. (**A**) WISH expression analysis of the neural progenitor marker *pax6a* in control or MCT8 morphant zebrafish embryos at 25hpf; *pax6a* was regulated in a context dependent manner by MTHs during zebrafish embryogenesis. Lateral images of the hindbrain and spinal cord in embryos are presented. (**B**) WISH expression analysis of the neural progenitor factor *neurod6b*. This gene was regulated by MTHs in the mid- and hindbrain. Lateral (upper panel) and dorsal images (lower panel) of the brain in embryos is presented. (**C**) WISH expression analysis of the *retinoic orphan receptor ab* (*rorab*). Regulation by MTHs occurred in the midbrain and eyes. Lateral (first panel) and dorsal images (second panel) of the brain in embryos are presented. Red arrowheads indicate the optic tectum. (**D**) WISH analysis of the expression of the inhibitory neuron marker, *gad1b*, showing that the development of inhibitory neurons was dependent on MTHs during zebrafish embryogenesis. Lateral and dorsal images of the brain (first and second panels) and lateral images of the spinal cord (lower panel) in embryos are presented. The red arrowheads indicate the midbrain-hindbrain boundary (MHB). ey – eye, fb – forebrain, hb-hindbrain, md – midbrain, nt-notochord. In all images the scale bars represent 100 μm.

Given that *her2* plays an important role in the generation and maintenance of neural progenitor cells in the brain and SC, the evidence suggests that these cells are under the regulation of MTHs during zebrafish embryogenesis. This evidence was further confirmed by down-regulation of *pax6a* and *neurod6b*, which are two important factors involved in neural progenitor cell differentiation and survival (Fig. 3A and B). At 25hpf expression of *pax6a* was down-regulated in MCT8 morphants in a cell-specific manner in the brain and SC (Fig. 3A). The down-regulation of *pax6a* was further observed at 48hpf (Supplementary Fig. 5A) and the hindbrain was the region of the brain most affected by absence of MTH (Fig. 3A and Supplementary Fig. 5A). The most affected expression domain of *pax6a* was the SC where most dorsal *pax6*-positive cells seemed to be more dependent on MTH signalling than the more ventral *pax6a*-positive cells (Fig. 3A and Supplementary Fig. 5A).

Further evidence that neural progenitors are a key target for MTHs during zebrafish embryogenesis was revealed by the results of WISH for *neurod6b* (Fig. 3B and Supplementary Fig. 5B). In MCT8 morphants at 25hpf the most anterior *neurod6b* positive-cells in the forebrain were dependent on MTHs but posterior cells lying near the FMB were not affected (Fig. 3B). This was also true at 48hpf (Supplementary Fig. 5B) when midbrain and hindbrain expression domains of *neurod6b* were totally dependent on MTHs whereas the forebrain domain was similar to that at 25hpf (Fig. 3B and Supplementary Fig. 5B). These results were further confirmed in rescued 25hpf embryos (Supplementary Fig. 8A and B).

Expression of the retinoic acid orphan receptor ab, *rorab*, which is involved in generation of midbrain neuron diversity, was severely affected in the midbrain of MCT8 morphants (red arrowhead in Fig. 3C). At 25hpf *rorab* was down-regulated in the midbrain of MCT8 morphants but increased at 48hpf (Fig. 3C and Supplementary Fig. 5C) and in the retina *rorab* expression was decreased although not as much as in the midbrain (Fig. 3C and Supplementary Fig. 5C). In common with the midbrain domain, in the retina *rorab* expression was lower at 48hpf than at 25hpf (Supplementary Fig. 5C and Fig. 3C). The loss of *rorab* was fully recovered in rescue 25hpf embryos (Supplementary Fig. 8C).

The expression of glutamate decarboxylase 1b (*gad1b*), which is only found in GABAergic neurons, was also dependent on MTHs (Fig. 3D and Supplementary Fig. 5D). The effect of MCT8 knockdown on *gad1b* expression was tissue/cell specific and the hindbrain was especially sensitive, with most *gad1b* positive cells being lost at both 25hpf and 48hpf (Fig. 3D and Supplementary Fig. 5D). Within the hindbrain at 25hpf anterior *gad1b*-expressing cells were less dependent on MTHs and at 48hpf most ventral *gad1b* positive cells were lost, whereas dorsal cells were mostly retained (Supplementary Fig. 5D). In the SC most *gad1b* positive cells were retained but they had a modified spatial organization relative to the control (Fig. 3D and Supplementary Fig. 5D). Nonetheless, at 48hpf almost all *gad1b* positive cells were lost in the SC of MCT8 morphants and only a few ventrally located cells still expressed *gad1b* albeit at very low levels (Supplementary Fig. 5D). These observations were further confirmed at 25hpf in rescue embryos (Supplementary Fig. 8D).

### MTHs are involved in angiogenic development during late embryogenesis

We have previously demonstrated that MTHs are important for angiogenesis in zebrafish during development, namely in the blood-hindbrain-barrier development^15^. We further explored this observation by selecting differentially expressed genes involved in angiogenesis and analysed their expression by WISH in MCT8 morphants. Genes involved in angiogenesis were much less represented in the MCT8 morphant transcriptome probably due to the rudimentary development of the vascular system at 25hpf (log2[fold-change] values). We looked into the expression of *flt4* (0.431) given its role in vascularization.

**Figure 4:**
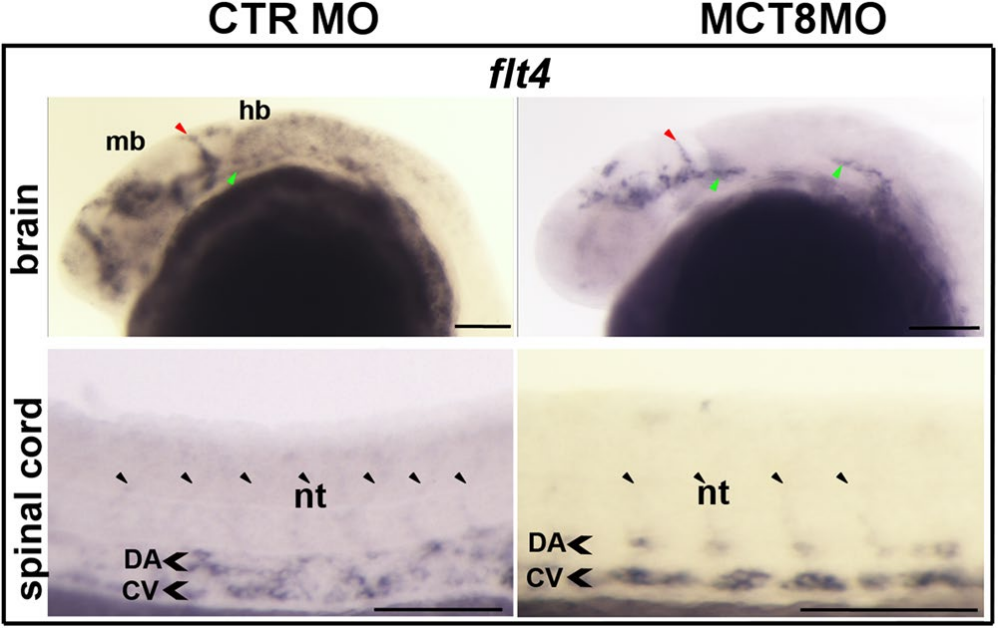
MTHs were important in angiogenesis during zebrafish development. A comparison between control and MCT8 morphant zebrafish embryos at 25hpf is presented. (**A**). WISH expression analysis of *flt4* showed that MTHs regulate *flt4* expression in a context dependent manner. Lateral images of the head (first panel) and the trunk (second panel) are shown. The red arrowheads indicate the mid-central vein, the green arrowheads indicate the primordial hindbrain channels and the black arrowheads indicate the intersegmental vessels. CV - Cardinal vein, DA - Dorsal artery, hb – hindbrain, mb – midbrain, nt - notochord. In all the images the scale bar represents 100 μm.

The whole body transcriptome analysis revealed that FMS-related tyrosine kinase 4 (flt4) was up-regulated in the MCT8 morphants (log2fold 0.431, p < 0.01, FDR < 0.05). However, *in situ* analysis revealed that the transcriptional response of *flt4* in MCT8 morphants was not generalized but rather context specific (Fig. 4 and Supplementary Fig. 6). At 25hpf in the head region of MCT8 morphants, *flt4* expression was lost in the hindbrain whereas in the developing body vasculature its expression was conserved (Fig. 4). Significant disruption of the head vascular structures was observed and the primordial hindbrain channel (green arrowhead in Fig. 4) was incompletely developed and the midbrain and forebrain structures were severely disrupted. In the trunk of 25hpf MCT8 morphants, intersegmental vessels (black arrowheads in Fig. 4) did not appear to be modified but expression in the dorsal artery (DA) and cardinal vein (CV) was slightly increased relative to control siblings (Fig. 4). At 48hpf there was a slight but observable increase in the expression of *flt4* in the head vasculature (Supplementary Fig. 6). The slight increase in *flt4* expression at 25hpf in the DA and CV of MCT8 morphants was still evident in 48hpf morphants (Fig. 4 and Supplementary Fig. 6). The effects of MCT8 knockdown on flt4 at 25hpf were fully recovered in rescue embryos (Supplementary Fig. 9).

## Discussion

The MCT8 transporter is extremely important for the appropriate regulation of intracellular TH levels. This has been clearly demonstrated by studies showing that MCT8 mutations affect transporter characteristics and consequently TH transport efficiency, giving rise to the phenotypic variability observed in AHDS patients^25,30,31^. Furthermore the function of MCT8 mutants is also dependent on the cellular context^31^. The only known function of MCT8 is facilitated cellular transport of iodothyronines across the plasma membrane and no other substrates have been described in vertebrates^32,33^ and a recent study confirmed that MCT8 lacks constitutive activity^34^. We have previously shown that the zebrafish MCT8 knockdown displayed locomotor, cellular and molecular changes congruent with the human neurological symptoms of AHDS^15^. Here, by characterising the transcriptome of zebrafish MCT8 morphants, we confirmed the importance of MTHs in regulation of key genetic pathways during zebrafish development. Our data strongly point to conservation of the MTH-dependent genetic mechanisms in central nervous system (CNS) development in zebrafish, mouse cell models^35–37^ and human patients of AHDS^38^.

The number of different genes affected by MTHs (>4500) and the fold-changes in expression of these genes, indicates that MTHs regulate a wide variety of processes during zebrafish development. In this study we did not established if the action of MTHs on target genes was direct or indirect. However, we identified the genes and pathways involved in zebrafish embryogenesis that were regulated by MTHs. Transcriptome and pathway analysis revealed that MTHs regulated an extensive array of cell signalling processes that in turn are involved in different cellular and developmental pathways. The observation that MTHs regulate genes in all Reactome mother categories (Fig. 1C) indicates the breadth of their action in developing zebrafish embryo.

Extracellular matrix organization (ECM) was the most populated Reactome mother category. The most striking phenotype in MCT8 knockdown zebrafish was linked to the function of MTHs on nervous system development where the ECM is a fundamental constituent and determinant for appropriate CNS function, as occurs in mammals^39^. The detailed mode and mechanisms of MTH regulation is still unknown. However, comparison of our data with other data on MTH function in vertebrate embryogenesis indicated a high level of conservation^17,40,41^. The developmental pathways regulated in zebrafish were common with those in mammals^36,37,39^, especially those related to CNS development, and further highlighted the importance of MTH signalling during vertebrate embryogenesis. In fact, a detailed comparison of the zebrafish MCT8 morphant transcriptome from the present study and T3-regulated genes in mice neural derived cells, revealed that 59.4% of the genes reported were common^35^.

MTHs regulated major development-related pathways like NOTCH, WNT and Hedgehog in a pathway specific manner during zebrafish development. In the NOTCH and Hedgehog sub-pathways MTHs regulated genes in almost all reactions, while in the WNT sub-pathways MTH regulated genes were more variable. WNT signalling^42^ has previously been linked to THs but less is known about the role of THs in the NOTCH pathway. In fact C17 cells expressing Trha or Trhb^36^ or primary mouse cerebrocortical cells treated with T3^37^ revealed up-regulation of the Notch1 and 4 receptor, Jag1, Hr and Hes6 expression, whereas in the zebrafish MCT8 morphants all these genes were down-regulated. This data indicates that the regulation of the NOTCH pathway by THs is a common feature of vertebrate CNS development.

Much of the cellular evidence collected from human patients with AHDS was complemented and supported by our zebrafish transcriptome dataset. Noticeably the majority of the TH dependent neuronal proteins with altered expression in humans^38^ where down-regulated (p < 0.01; FDR < 0.05) in the zebrafish MCT8 morphant transcriptomes, examples include: *neflb* (−0.94) *pvalb3* (−0.65) *pvalb4* (-1.05), *pvalb6* (-1.16), *pvalb7* (-1.08), *pvalb8* (-0.60), *pvalb9* (-0.57), *syp* (-0.35), *calb1* (-1.04) and *calb2b* (-0.63). These results support the notion that the underlying genetic mechanisms of AHDS have been conserved between humans and zebrafish morphants^15^.

Detailed spatial-temporal mapping of selected MTH-regulated genes during zebrafish embryogenesis revealed a role for MTHs in maintenance of specific neuronal progenitor populations, brain region definition, neuronal cell diversity generation and function and brain vascularization. We report for the first time that vertebrate DeltaA together with *her2*, *neurod6b* and *pax6a* genes were regulated by THs, suggesting they have a role in the maintenance of specific progenitor populations in the hindbrain and spinal cord and in generating cellular diversity in later embryonic neural development (this study and^15^). The results of the present study show that the MCT8 morphant embryos have problems both in brain development and function in common with human patients of AHDS.

In common with *wnt1* mutants^43^ the MCT8 morphants have a less defined midbrain-hindbrain boundary (MHB) and this seems to be contradictory to the broadening of the expression field of *wnt1* in MHB. Nonetheless, the field of expression of the WNT1 protein secretion regulator *wls* ^28^ in the MHB was also increased indicating that MTHs might regulate different post-transcriptional WNT-related cellular events in MHB development. Taken together the evidence suggests that the role of MTHs in MHB development might precede WNT1 function or that MTHs regulate cellular events that modulate WNT1 function. However it was not possible in the present study to establish if MTHs have a direct or indirect effect on WNT related genes or if MTH action on WNT signalling was cell-specific. The present data support a role for MTHs in hindbrain development in zebrafish as has been observed in mammals^1,2,17,44–46^ and corroborates studies in zebrafish development in which overexpression of *thraa* disrupted normal hindbrain development^13^.

One of the developmental pathways most affected by the lack of MTHs was the NOTCH pathway, which regulates the neural progenitor pool^47^. Notably, Dla and *her2* are key target genes by which MTHs affect neural progenitor pools. Given the dependence of Delta-Notch signalling for *her2* expression^48^ it was not possible to establish in the present study if MTHs acted directly on delta expression, or via *her2* or both. Notably the mammalian homolog of zebrafish *her2*, *Hr*, is a direct target of THs in mice^36,37,39,46^. Nonetheless, the transcriptome data at 25hpf revealed up-regulation of the *her2*-regulated cell cycle inhibitor genes *cdkn1a* (0.92- log2fold increase, p < 0.01, FDR < 0.0001) and *cdkn1ba* (0.94- log2fold increase, p < 0.01, FDR < 0.0001), which was consistent with a decrease in the maintenance of the *her2* positive neural progenitors^48^. Our data also strongly suggest that MTHs have a cell context specific effect on *her2* expression and the neural progenitor pools given that the hindbrain and SC seem to be much more sensitive to the lack of MTH signalling than the midbrain and forebrain (Fig. 2D and Supplementary Fig. 4D). The finding that Dla was also affected might indicate further imbalance in the neuron/progenitor cell ratios that suggests a diminishing neural stem cell population in MCT8 morphants. Previously we have reported that hindbrain *pax8* positive inhibitory neurons were lost in the hindbrain and SC of MCT8 morphant embryos^15^. The present data leads us to hypothesize that MTHs act upon specific neural progenitor populations that originate *pax8* positive-inhibitory neurons. The loss of MTHs in the MCT8 morphants led to abnormal distribution of *pax2a* neurons in the hindbrain and SC^17^. This hypothesis was supported by the finding that *gad1b* expression was not entirely lost in the hindbrain and SC of MCT8 morphants (both *pax2a* and *pax8* neurons were *gad1b*-positive^49^) but its distribution was abnormal and resembled *pax2a* ^15^, especially in the SC at 25hpf. The action of MTHs on the progenitor cell population was further supported by the downregulation, in the MCT8 morphant transcriptome, of 2 key genes (*her2* and *ascl1*) that are involved in the development of GABAergic neurons in zebrafish^50,51^.

The fact that neural progenitor markers such as *pax6a* and *neurod6b* were more dependent on MTHs in the hindbrain than in the midbrain and forebrain, suggested that the development of this brain region was more dependent on MTHs (Fig. 3A and B). Given that *pax6a* and *neurod6b* are involved in the generation/maintenance of neural progenitors that give rise to glutamatergic neurons^52–54^ it is very likely that at least some of these neuron populations were lost due to impaired MTHs signalling in the MCT8 MO zebrafish. The results in zebrafish contribute to explain the observed psychomotor retardation consequences of AHDS in human patients^21,55,56^. The loss of neural stem cell/progenitor cells in a very early stage of development may explain the lack of neurological improvement of AHDS patients after treatment with TH therapies (reviewed^57^).

The transcriptional regulation of *rorab* by MTHs indicates that they are not only involved in regulating brain region specialization but likely also generate neural cell diversity and specialized brain functions. In mice and chicken RORa is involved in Purkinje cell and hindbrain development and is under the regulation of THs via thra1^44,58,59^. However, in zebrafish *rorab* is absent from the hindbrain and is not involved in Purkinje cells development^45^. Nonetheless, the expression of *rorab* in both the eyes and optic tectum strongly suggests that *rorab* is involved in development of vision and visual signal processing in zebrafish. It is not known if THs regulate zebrafish *rorab* expression directly but the expression of *thraa* and *thrab* in the optic tectum during zebrafish embryogenesis strongly suggests that like in mice and chicken^44,58,59^, *rorab* was regulated by MTHs. Given these results it is likely that patients with AHDS may also suffer from vision related impairment.

The results of our study strongly suggest a role for MTHs in brain vascularization. For example, expression of *flt4* was up-regulated in MCT8 morphants and *rspoI*, which is involved in wnt-vegfc-flt4 signalling during zebrafish angiogenesis^60^, also had an increased expression in RNA-seq (Log2fold increase 1.06, p < 0.01, FDR = 0.00005). The increase in both these genes in the MCT8 morphants suggests that MTHs may modulate rspo1/wnt-vegfc-flt4 signalling during development of blood vessels. However, it remains unclear how MTHs regulate rspo1/wnt-vegfc-flt4 signalling and more work will be necessary to further clarify these observations.

Taken together our results show that MTHs: (1) participated in a variety of developmental signalling cascades during zebrafish embryogenesis; (2) were fundamental for late neural development, including brain compartmentalization, neuronal cell diversity, neuronal stem cell pool maintenance and function and; (3) were most likely involved in the vascularization of the brain. As a whole the results of our study suggest that MTHs may integrate and coordinate the action of different signalling pathways and the processes that underpin the development of a fully functional organism. Given that: (1) the zebrafish MCT8 morphant had a similar set of T3-responsive genes to those found in in mice neural derived cells^35–37^ and human AHDS patients^38^ and (2) only in zebrafish was the locomotor phenotype typical of AHDS replicated, we propose that the zebrafish MCT8 knockdown model represents a suitable vertebrate experimental model to study human AHDS.

## Methods

### Animal culture, embryo generation and microinjection of control and MCT8 MO

Adult zebrafish (AB strain) were maintained at 28 °C in a 14:10 (h:h) light:dark cycle and allowed to mate naturally in mating boxes as previously described^15^. Embryos were immediately collected after fertilization and microinjected at the 1-2-cell stage with 1nL of MO solution containing either 0.8pmol CTR MO or MCT8 MO as previously described^15^. The MCT8 MO has been fully validated in our previous publication^15^. The mortality of microinjected embryos was verified 10 hpf and morphant embryos were sampled at 25hpf when the morphant phenotype was clear. Seven independent experiments were carried out and each constituted a biological replicate. All sampling was carried out on live animals using the morphological characteristics of control embryos at 28 °C and followed the Kimmel classification scheme for staging zebrafish embryos^61^.

All experiments were approved by the ethical committee of CCMAR and were in accordance with the regulation of Directive 2010/63/EU (EU, 2010).

### Isolation of total RNA from experimental embryos

For each biological replicate (n = 7) experimental embryos (MCT8 MO and CTR MO embryos) at 25hpf were dechorinated, and pooled (~50 embryos per replicate) and preserved in RNAlater reagent (Sigma-Aldrich) and stored at -20 °C until use.

Collected embryos were removed from RNAlater reagent and homogenised with a glass homogeniser and RNA extracted using an OMEGA Total RNA extraction kit I as described by the manufacturer. Total RNA was subjected to DNAse treatment using an Ambion Turbo DNAse kit as described by the manufacturer. The quality (RIN > 8) and quantity of total RNA was verified with a BIO-RAD Experion Total RNA chip following the manufacturer’s instructions. Ten micrograms of total RNA per sample was shipped on dry-ice to the Oklahoma Medical Research Foundation NGS core facility for Illumina RNA-seq sequencing (USA). One microgram of total RNA per sample was amplified using a TrueSeq stranded pair-end Illumina kit following the standard protocol. Sequencing of mRNA was conducted on an Illumina HiSeq instrument and 50 base paired end reads generated. cDNA libraries from each of the 14 biological samples (n = 7 controls and n = 7 MCT8 MO zebrafish 25hpf) was randomized and then sequenced in two lanes of the HiSeq instrument, following an experimental balanced block design.

### Transcriptome assembly, annotation and analysis

Quality control of raw reads and editing was performed with Trimgalore wrapper script version 0.3.3 (bioinformatics.babraham.ac.uk/projects/trim_galore) producing simple descriptive statistics and edited reads. Edited and cleaned reads were mapped to the zebrafish reference genome (zfish_GRCz10.80) with Tophat version 2.0.13^62^ using the sequence aligner bowtie2 version 2.2.4^63^, and the fasta and respective annotation file (.gtf) were downloaded from the Ensembl Genome Browser (http://ensembl.org). Assembled transcripts and gene transcript estimated abundance was generated in the Cufflinks workflow, version 2.2.1^62^ and used to establish differential gene expression using EdgeR version 3.12.1^62,64^. Expression plots were designed using CummeRbund version 2.16.0. Gene ontology (GO) analysis was performed using the enrichment analysis tool of the Gene Ontology Consortium^65^, and enriched GO terms were summarized with ReViGO (http://revigo.irb.hr)^66^ by removing redundant GO terms.

Pathway analysis was carried out using the Reactome V58 (www.reactome.org)^26^ curated pathway resource. Mapping of differentially expressed genes and their expression fold change between control and MCT8 MOs was established using human pathway data. Pathways under the regulation of MTH were taken to be significant at p < 0.05.

### *In situ* validation of RNA-seq transcriptome analysis

The *neurod6b*; *wnt1; gad1b;* and *flt4* plasmids were kindly provided by Professors Monte Westerfield; Nobuyuki Ithoh and Ayumi Miyake; Brant Weinstein; Wolfgang Driever; and Schulte-Merker, respectively.

The *pax6a*, *rorab*, *her2*, and *wls* primers (Table 1) were designed using as template the sequences from the assembled transcriptome. Isolation of the cDNA of selected genes was carried out using a Thermo DreamTaq PCR kit following the manufacturers recommendation, and the amplified fragment isolated by agarose gel band extraction after electrophoresis and cloned into a pGemT easy vector as described by the manufacturer (Promega). Isolated plasmid DNA was sequenced to confirm the identity of each clone by querying the NCBI nr nucleotide database using blastn.

**Table 1:** Primer sequences used to isolate zebrafish *pax6a*, *her2*, *rorab* and *wls* cDNA.

Gene	Forward primer (5′-3′)	Reverse primer (5′-3′)
*pax6a*	AGGCTGTTGGAACTATGCCTC	CGTCGCGTTCTCACTGTAGTC
*her2*	CACACACGCGAGCTCTGACAGC	CACCTCTGCAGGCTACACATCTC
*rorab*	CTCAGATTGAGAGTATTCCCTG	CTCTACTCTGGTCTTCTC CTG
*wls*	CTGGTCCACCTGGATGCCGTG	GGGTAGAGGTTATTCTTGAGC

One-cell stage embryos microinjected with either 0.8 pmol of either CTR MO (GeneTools) or MCT8 MO^15^ were fixed at 25 hpf and 48 hpf in ice-cold 4%PFA/PBS overnight at 4 °C. The following day the samples were washed and transferred into 100% methanol and kept at -20 °C until used for WISH. WISH was carried out as previously described^15^. For image analysis samples were transferred to 100% glycerol and photographed under a stereoscope (Olympus) coupled to an OPTICA 3.0 digital colour camera. At least ten animals per stage and experimental condition were analysed.

Rescue experiments were also carried out and one-cell stage embryos were injected with either 0.8 pmol CTR MO, 0.8 pmol MCT8 MO or 0.8 pmol MCT8 MO + 100 pg mutated MCT8 mRNA as previously described^15^. WISH analysis of the genes of interest was carried out on rescue embryos in order to fully validate MCT8 MO specificity in line with already published validations^15^.

### Immunohistochemistry

Fluorescent immunohistochemistry against zebrafish Dla was carried out as previously described^29^. The primary antibody fluorescent labelling was carried out using a goat anti-mouse IgG-CF594 (Sigma) anti-serum (1/400). Fluorescent imaging of the hindbrain and SC was carried out using a Zeiss Z2 microscope coupled to a Zeiss HRm digital camera. Hindbrain image stacks were deconvoluted in SCI Huygens software. Hindbrain and SC maximum projections were generated in FIJI^67^.

### Data availability

All RNA-seq data generated is available through the BioProjects portal at the NCBI website with the accession number PRJNA381309.

## Electronic supplementary material

Supplementary Figures
