# Supplementary material for: Transcriptomics reveal an integrative role for maternal thyroid hormones during zebrafish embryogenesis
Source: Sci Rep. 2017 Nov 30;7:16657. doi: 10.1038/s41598-017-16951-9 (PMC5709499; doi:10.1038/s41598-017-16951-9)
Supplement: Supplementary file 1 — Supplementary Figures [file 41598_2017_16951_MOESM1_ESM.pdf]

Transcriptomics reveal an integrative role for maternal thyroid hormones during zebrafish embryogenesis

Nadia Silva, Bruno Louro, Marlene Trindade, Deborah M. Power, Marco A. Campinho*

Comparative Endocrinology and Integrative Biology Group, Centre for Marine Sciences (CCMAR), Universidade do Algarve, Faro Portugal

*Correspondance to macampinho@ualg.pt

Supplementary Figure 1

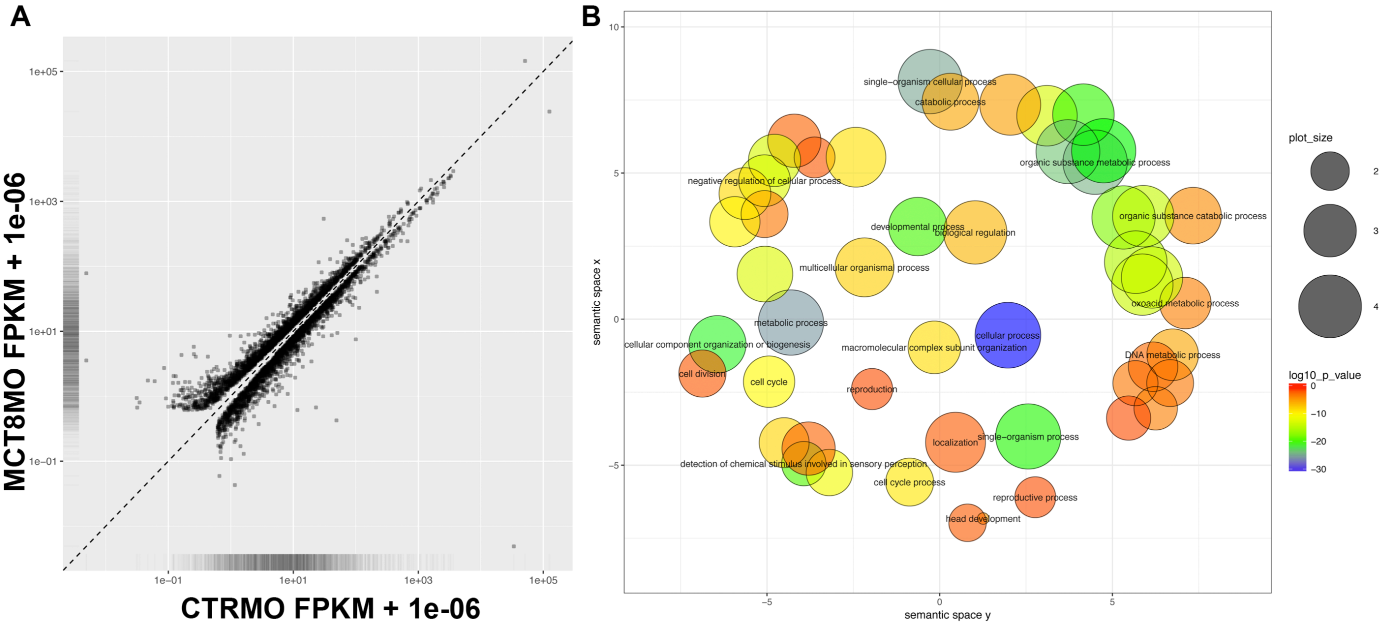

Supplementary Figure 2

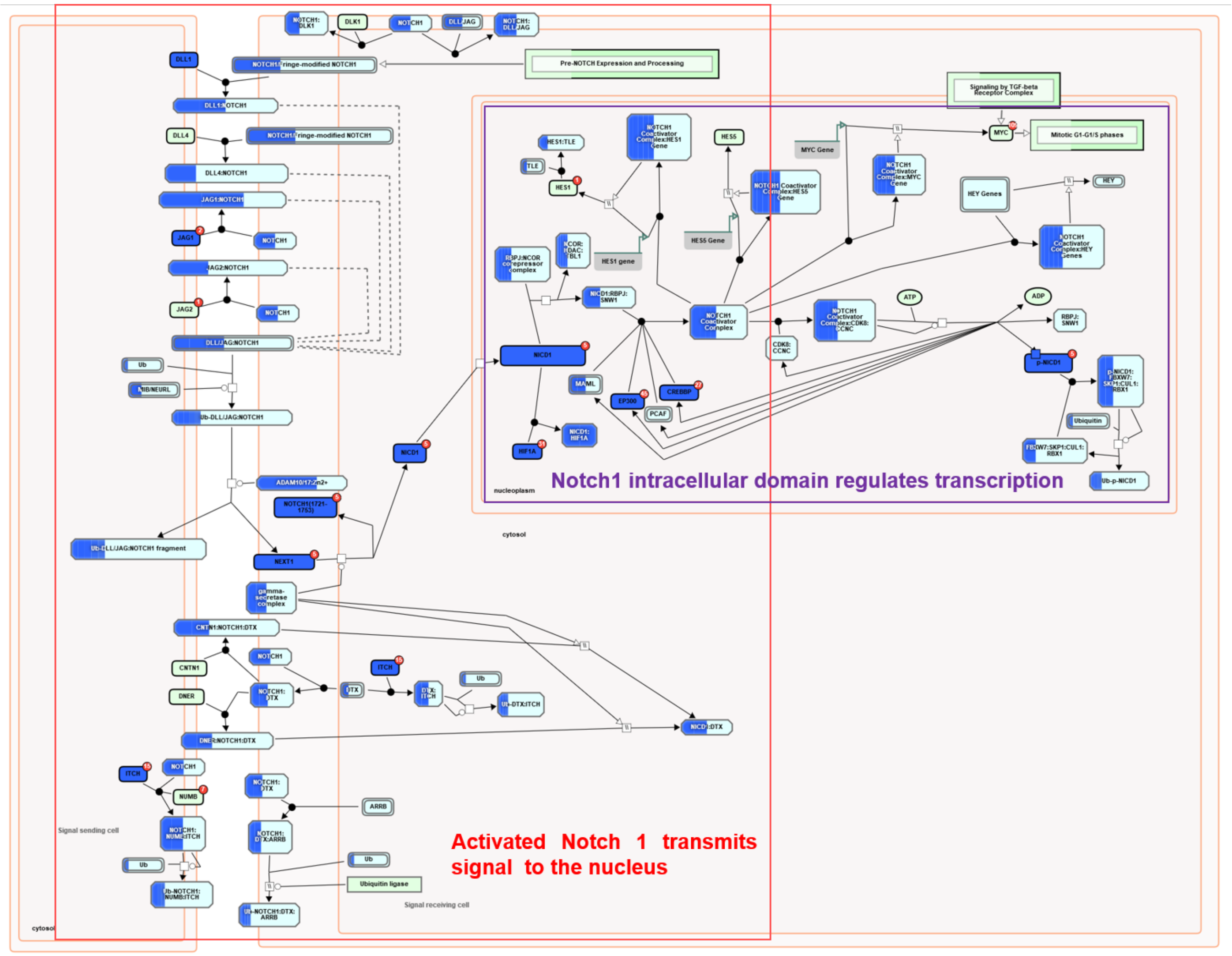

Supplementary Figure 3

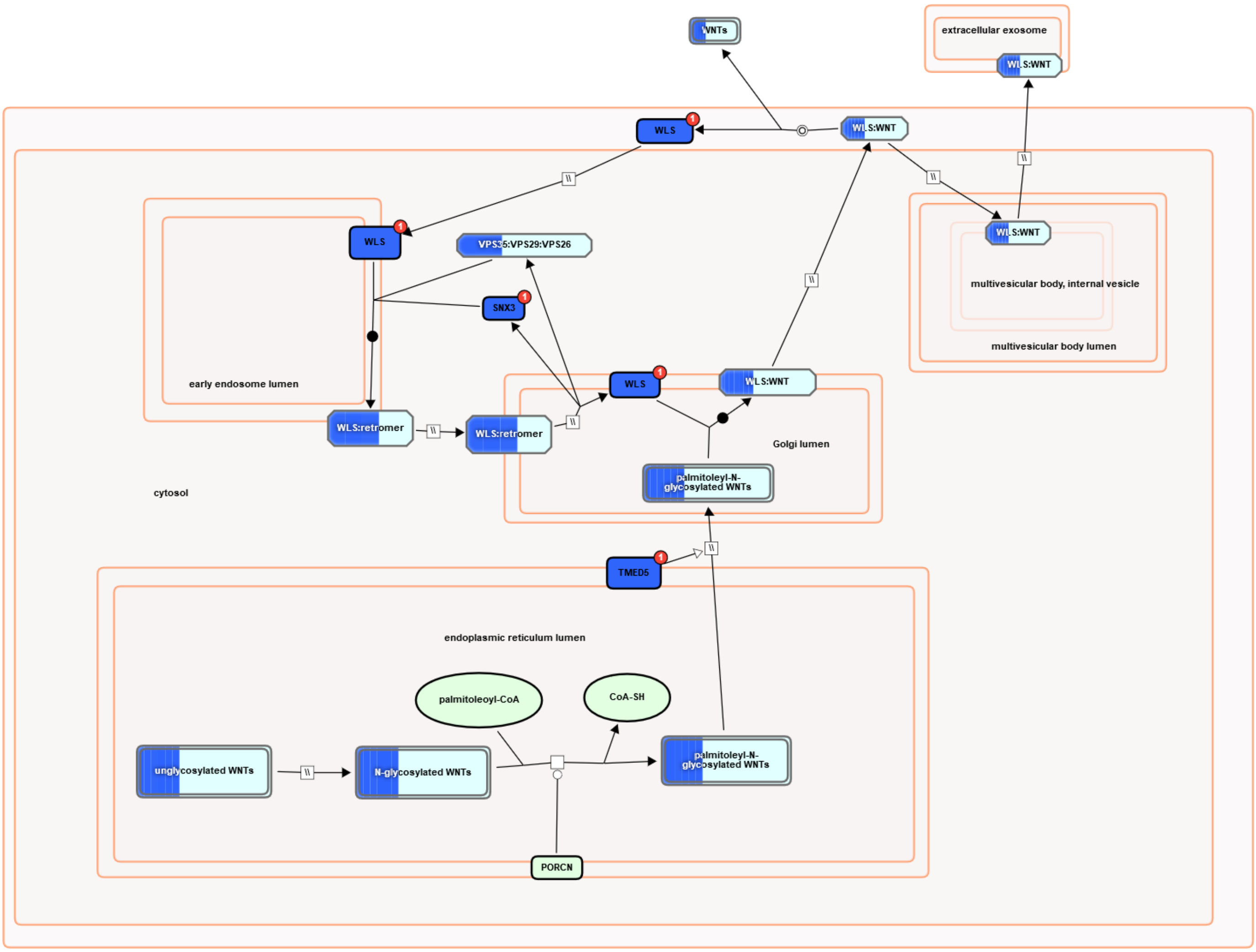

Supplementary Figure 4

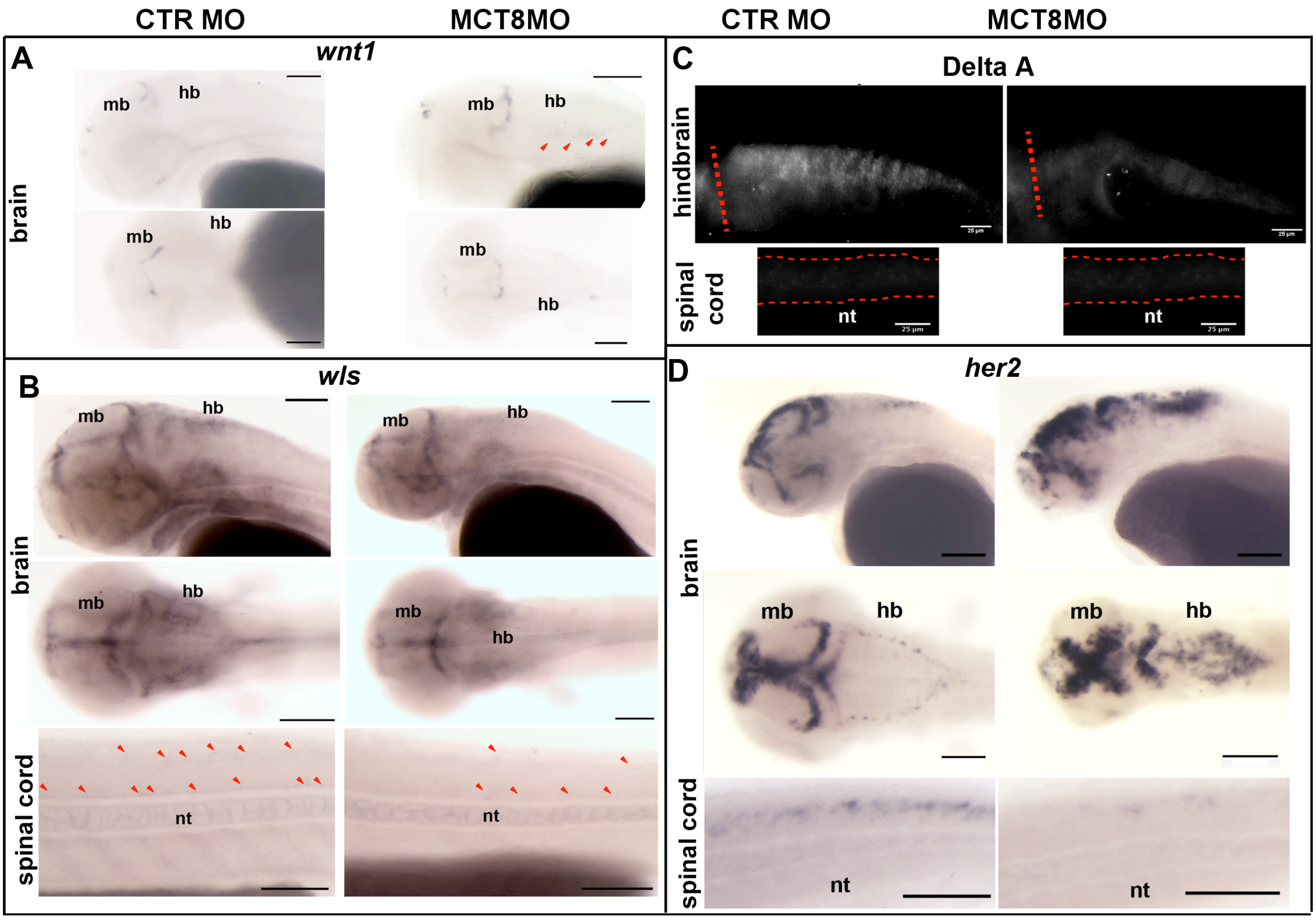

Supplementary Figure 5

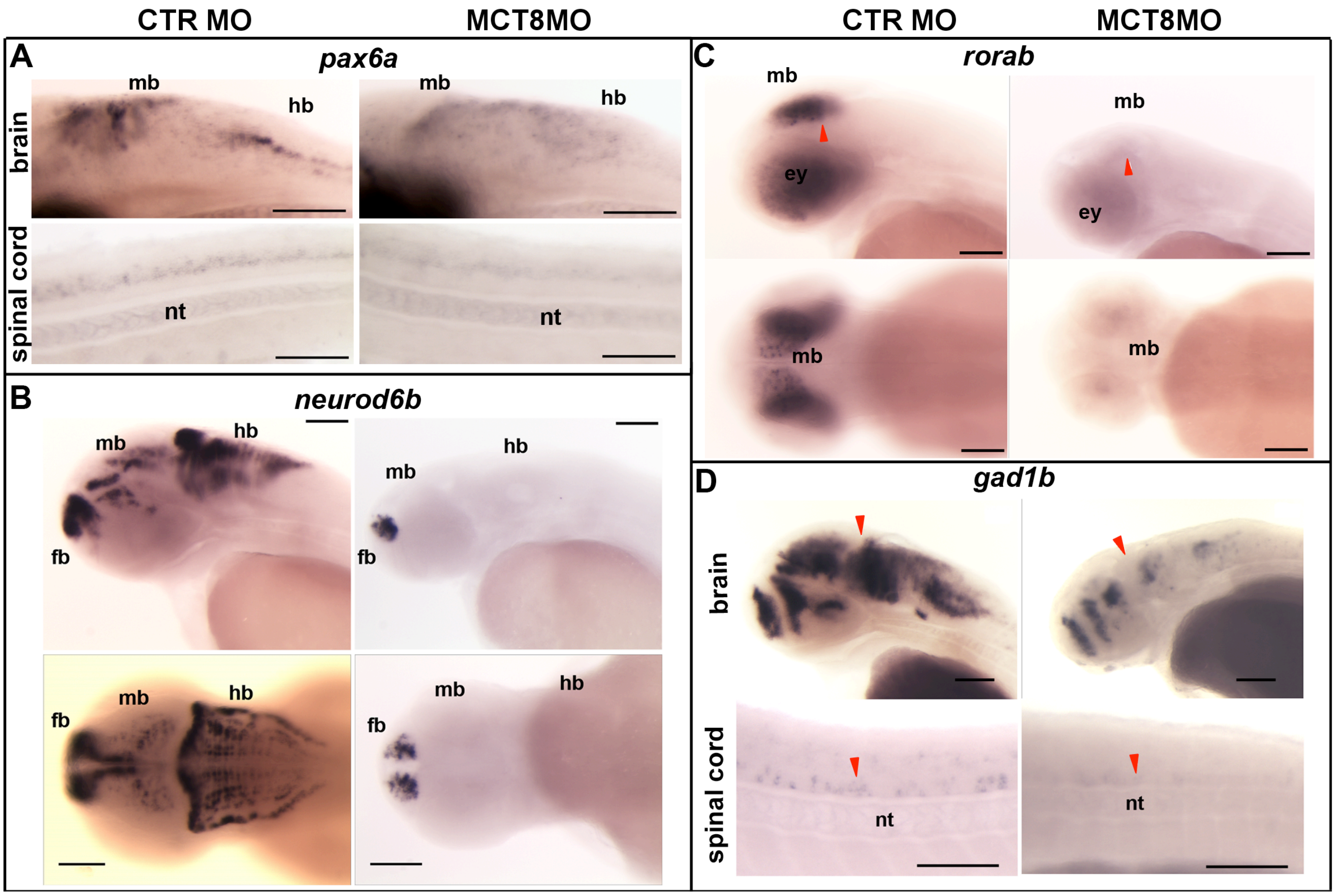

Supplementary Figure 6

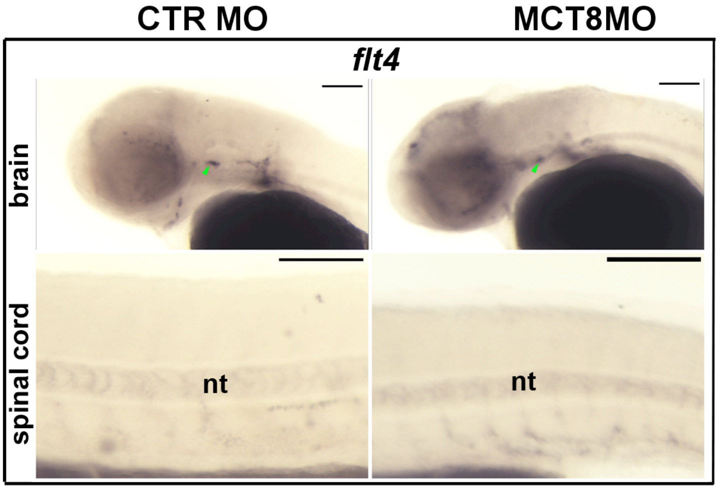

Supplementary Figure 7

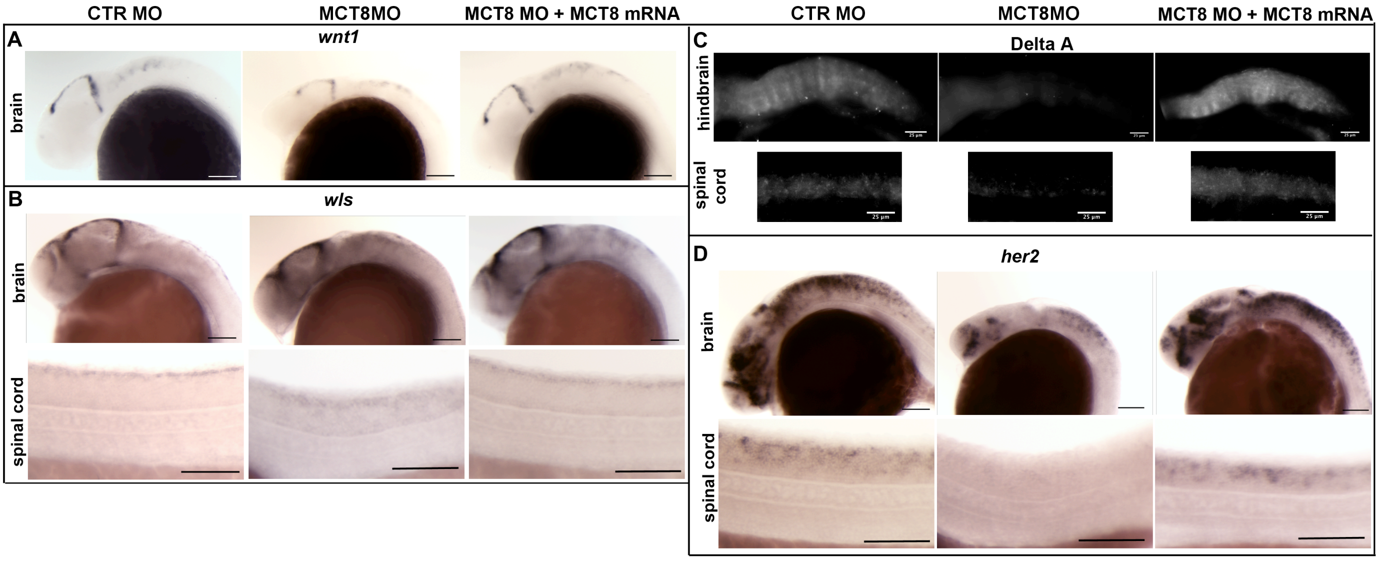

Supplementary Figure 8

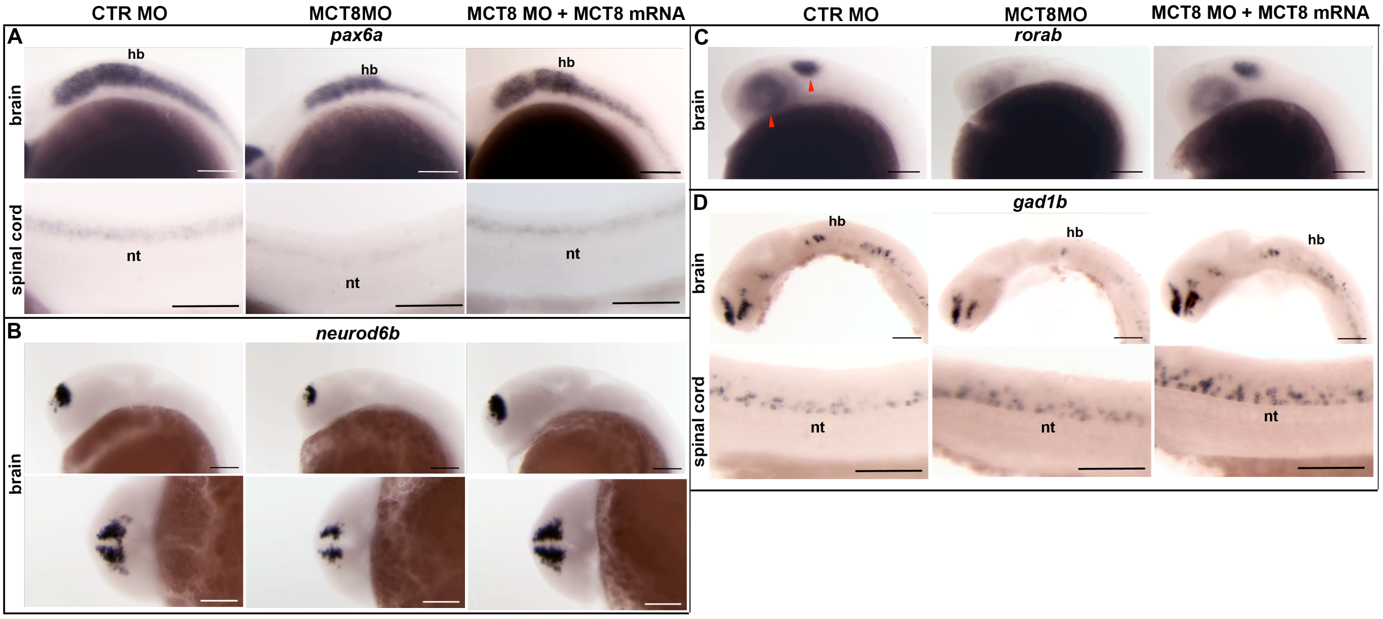

Supplementary Figure 9

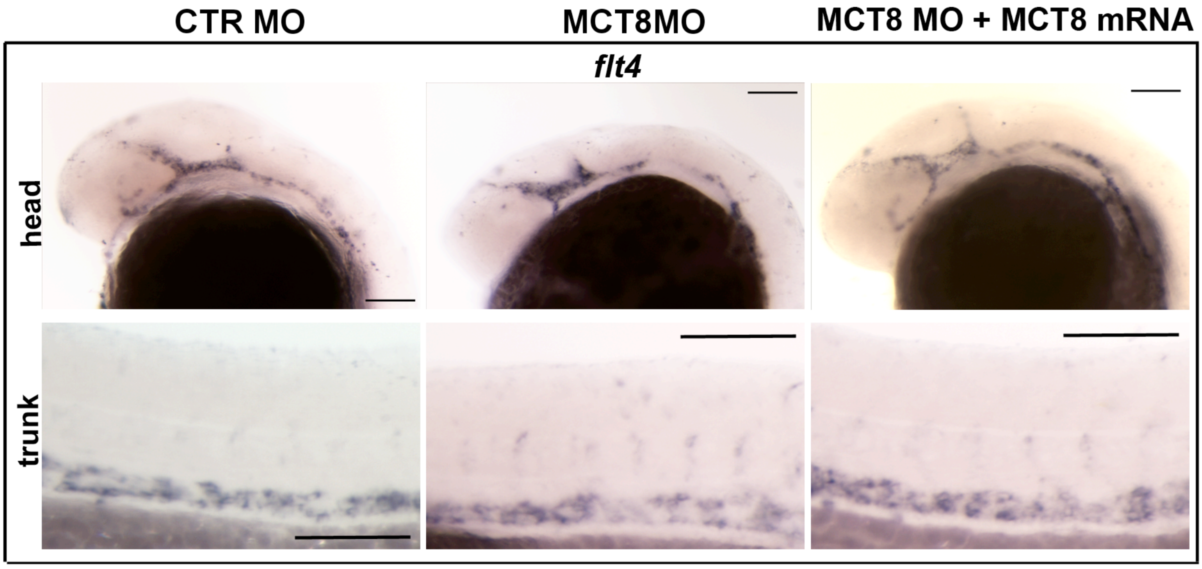

Supplementary Table 1. Enriched statistical significant non-redundant GO categories for Molecular and Biological Function used for ReViGO. Table presents plot sizes of each GO category and respective log10 p-value.

**Molecular Function**			
GOterm_ID	description	plot_size	log10 p-value
GO:0000988	protein binding transcription factor activity	2.233	-4.51
GO:0000989	transcription factor binding transcription factor activity	2.233	-4.3089
GO:0003682	chromatin binding	2.338	-7.153
GO:0003824	catalytic activity	4.105	-13.5952
GO:0004984	olfactory receptor activity	2.354	-17.7328
GO:0005488	binding	4.332	-35.0472
GO:0016740	transferase activity	3.7	-2.5884
GO:0060089	molecular transducer activity	3.471	-4.556
GO:0042802	identical protein binding	2.053	-2.5214
GO:0003723	RNA binding	2.977	-4.3536
GO:0048037	cofactor binding	2.728	-1.4976
GO:0005515	protein binding	3.57	-33.4672
GO:0044877	macromolecular complex binding	3.358	-7.3161
GO:1901363	heterocyclic compound binding	4.107	-9.466
GO:0097367	carbohydrate derivative binding	3.716	-1.8794
GO:0043168	anion binding	3.762	-3.8356
GO:0036094	small molecule binding	3.801	-6.5361
GO:0097159	organic cyclic compound binding	4.108	-9.4535
GO:0043167	ion binding	4.138	-6.58
GO:0003676	nucleic acid binding	3.862	-6.4225
GO:0044822	poly(A) RNA binding	1.74	-4.3883
GO:0000975	regulatory region DNA binding	2.057	-2.3788
GO:0001067	regulatory region nucleic acid binding	2.057	-2.3516
GO:0016787	hydrolase activity	3.685	-1.757
GO:0000166	nucleotide binding	3.789	-4.5735
GO:1901265	nucleoside phosphate binding	3.789	-4.5544
GO:0003677	DNA binding	3.514	-1.4921
Biological Function			
GOterm_ID	description	plot_size	log10 p-value
GO:0000003	reproduction	2.152	-1.4123
GO:0008152	metabolic process	4.275	-25.9281
GO:0009987	cellular process	4.324	-31.4001
GO:0022414	reproductive process	2.137	-1.4522
GO:0032501	multicellular organismal process	3.592	-7.821
GO:0032502	developmental process	3.536	-20.0888
GO:0044699	single-organism process	4.254	-20.767
GO:0048523	negative regulation of cellular process	2.929	-12.5186
GO:0050907	detection of chemical stimulus involved in sensory perception	2.362	-19.7932

GO:0051179	localization	3.699	-1.9245
GO:0065007	biological regulation	4.051	-6.3737
GO:0071840	cellular component organization or biogenesis	3.439	-21.3251
GO:0006259	DNA metabolic process	2.92	-5.4855
GO:0043933	macromolecular complex subunit organization	3.011	-8.699
GO:0060322	head development	1.23	-5.007
GO:0007049	cell cycle	2.903	-10.2933
GO:0071704	organic substance metabolic process	4.165	-24.6402
GO:0051301	cell division	2.568	-1.7471
GO:0022402	cell cycle process	2.621	-9.2013
GO:0044763	single-organism cellular process	4.16	-25.2668
GO:0009056	catabolic process	3.388	-5.5867
GO:1901575	organic substance catabolic process	3.369	-3.9469
GO:0043436	oxoacid metabolic process	2.911	-3.5702
GO:0009058	biosynthetic process	3.755	-13.0937
GO:0042127	regulation of cell proliferation	2.137	-1.7352
GO:0006807	nitrogen compound metabolic process	3.866	-20.4737
GO:0010941	regulation of cell death	2.553	-3.1244
GO:0065008	regulation of biological quality	3.065	-2.1029
GO:0048519	negative regulation of biological process	2.975	-13.0872
GO:0048518	positive regulation of biological process	2.938	-8.3116
GO:0006457	protein folding	2.417	-3.2725
GO:0044710	single-organism metabolic process	3.803	-5.1296
GO:0048522	positive regulation of cellular process	2.816	-9.9318
GO:0051606	detection of stimulus	2.511	-11.0237
GO:0043170	macromolecule metabolic process	4.046	-18.0841
GO:0033554	cellular response to stress	2.782	-8.0585
GO:0044237	cellular metabolic process	4.117	-23.7282
GO:0019222	regulation of metabolic process	3.675	-8.9431
GO:0044238	primary metabolic process	4.157	-20.9355
GO:0006950	response to stress	3.066	-2.2083
GO:0003006	developmental process involved in reproduction	1.94	-1.9586
GO:1901360	organic cyclic compound metabolic process	3.848	-14.1871
GO:0018193	peptidyl-amino acid modification	2.747	-2.5072
GO:0007186	G-protein coupled receptor signaling pathway	3.284	-12.8268
GO:0044260	cellular macromolecule metabolic process	3.974	-15.3862
GO:0006260	DNA replication	2.303	-3.4225
GO:0046483	heterocycle metabolic process	3.841	-13.9747
GO:0006725	cellular aromatic compound metabolic process	3.841	-13.7496
GO:0043414	macromolecule methylation	2.356	-1.3251
GO:0097659	nucleic acid-templated transcription	2.548	-3.0726

Supplementary Table 2. Most populated REACTOME pathways by MTHs-regulated genes.

Pathway identifier	Pathway name	#Entities found	#Entities total	% of MTHs-regulated genes in Reactome categories
**R-HSA-428542**	Regulation of Commissural axon pathfinding by Slit and Robo	4	4	100
**R-HSA-3304347**	Loss of Function of SMAD4 in Cancer	3	3	100
**R-HSA-141424**	Amplification of signal from the kinetochores	2	2	100
**R-HSA-194306**	Neurophilin interactions with VEGF and VEGFR	3	4	75
**R-HSA-1980148**	Signalling by NOTCH3	7	12	58
**R-HSA-3238698**	Regulation of WNT ligand biogenesis and trafficking	10	28	36
**R-HSA-1980143**	Signalling by NOTCH1	26	82	32
**R-HSA-1474244**	Extracellular matrix organization	101	320	31
**R-HSA-1912422**	Pre-NOTCH Expression and Processing	20	76	26
**R-HSA-5358346**	Hedgehog ligand biogenesis	16	72	22
**R-HSA-5610787**	Hedgehog 'off' state	26	122	21
**R-HSA-5632684**	Hedgehog 'on' state	18	90	20
**R-HSA-201681**	TCF dependent signalling in response to WNT	41	215	19
**R-HSA-3858494**	Beta-catenin independent WNT signalling	31	163	19
**R-HSA-195253**	Degradation of beta-catenin by the destruction complex	14	89	16

Supplementary Figure 1. Zebrafish MTHs-dependent transcriptome at 25hpf. A) FPKM pairwise Scatter plot of differential expressed genes between control and MCT8 morphants at 25hpf ($p<0.01$; FDR<0.05). B) Biological process GO enrichment analysis slimmed with semantic clustered with REVIGO, Circle size represent frequency of GO terms and color scale is representative of log10 P-value.

Supplementary Figure 2. Reactome human pathway for Activated Notch 1 signalling. In the first part of the pathway, the activated Notch 1 transmits signal to the nucleus, 42% of the entities involved are modified by the lack of maternal thyroid hormone. Inside the nucleoplasm Notch 1 intracellular domain regulates the transcription of a series of target genes, here the outcome of mct8 “knock-down” is reflected in a change in expression of 30% of the entities involved. Proteins or protein complexes highlighted in blue correspond to differentially expressed genes in the maternal thyroid hormone dataset. For detailed diagram key please refer to Reactome (http://www.reactome.org/PathwayBrowser/#/R-HSA-1980143).

Supplementary Figure 3. Reactome human pathway for WNT ligand biogenesis and trafficking. Besides having an effect in the expression of several WNT ligands, MCT8 zebrafish morphants are show differences in proteins involved in different aspects of WNT trafficking and release form expressing cell. Proteins or protein complexes highlighted in blue correspond to differentially expressed genes in the maternal thyroid hormone dataset. For detailed diagram key please refer to Reactome (http://www.reactome.org/PathwayBrowser/#/R-HSA-3238698).

Supplementary Figure 4. MTHs regulate WNT and NOTCH pathway genes during zebrafish embryogenesis. WISH expression of differentially expressed genes ($p<0.01$; FDR 5%) and comparison between control and MCT8 morphant zebrafish embryos at 48hpf. (A)WISH expression analysis of *wnt1*. Lateral (first panel) and dorsal (second panel) images of brain in control and MCT8 morphant zebrafish embryos at 48hpf. Red arrows denote *wnt1* positive cells I the ventral hindbrain. (B) WNT1 protein secretion regulator - *wls* WISH expression, lateral (first panel) and dorsal (second panel) images of brain and lateral images of spinal cord in control and MCT8 morphant zebrafish embryos at 48hpf. Red arrows denote *wls*-expressing cells in the spinal cord. (C) Fluorescent WIHC expression analysis of DeltaA NOTCH ligand in

hindbrain and spinal cord of control and MCT8 morphant embryos at 48hpf. (D) WISH expression analysis of of NOTCH pathway target gene *her2* in control and MCT8 morphant zebrafish embryos at 48hpf. Lateral and dorsal images of brain (first and second panels) of analysed embryos are presented and lateral images of spinal cord are shown (lower panel). hb-hindbrain; mb-midbrain; nt-notochord In A,B,D scale bar represents 100μm. In C scale bar represents 25μm.

Supplementary Figure 5. Expression of differentially expressed genes ($p<0.01$; FDR 5%) involved in zebrafish neural development. Comparison between control and MCT8 morphant zebrafish embryos at 48hpf. (A) WISH expression analysis of neural progenitor marker *pax6a, this gene* is regulated in a context dependent manner by MTHs during zebrafish embryogenesis. Lateral images of the hindbrain and spinal cord of analysed embryos are presented (B) WISH expression analysis of neural progenitor factor *neurod6b*. This gene is regulated by MTH in the mid- and hindbrain. Lateral (upper panel) and dorsal images (lower panel) of the brain of analysed embryos is presented. hb-hindbrain, (C) WISH expression analysis of Retinoic orphan receptor ab (*rorab*). Regulation by MTH occurs in the midbrain and eyes. Lateral (first panel) and dorsal images (second panel) of the brain of analysed embryos are presented. Red arrowheads indicate the optic tectum. (D) WISH analysis of expression of inhibitory neuron marker *gad1b*, showing that inhibitory neurons development is dependent of MTHs in zebrafish embryogenesis. Lateral and dorsal images of brain (first and second panels) of analysed embryos are presented and lateral images of spinal cord are shown (lower panel). Red arrowhead indicates the midbrain-hindbrain boundary (MHB). nt-notochord, md – midbrain, fb – forebrain, ey – eye. In all images scale bar represents 100μm.

Supplementary Figure 6. MTHs are important for angiogenesis in zebrafish during development, comparison between control and MCT8 morphant zebrafish embryos at 48hpf (A). WISH expression analysis of *flt4* shows that MTHs regulate *flt4* expression in a context dependent manner. Lateral images of the head (first panel) and trunk (second panel) are shown. Green arrowhead depicts primordial hindbrain channels. In all images bar represents 100μm.

Supplementary Figure 7. Exogenous supply of MCT8 mRNA recovered the expression of genes affected by MCT8 MO to control levels in 25hpf zebrafish. (A) WISH expression analysis of *wnt1*. Lateral images of brain in control, MCT8 morphant and co-injection of MCT8 mRNA and MCT8 morpholino in zebrafish embryos at 25hpf. (B) WNT1 protein secretion regulator - *wls* WISH expression, lateral images of brain and lateral images of spinal cord in control, MCT8 morphant and co-injection of MCT8 mRNA and MCT8 morpholino in zebrafish embryos at 25hpf. (C) Fluorescent WIHC expression analysis of DeltaA NOTCH ligand in hindbrain and spinal cord of control, MCT8 morphant and co-injection of MCT8 mRNA and MCT8 morpholino in embryos at 25hpf. (D) WISH expression analysis of of NOTCH pathway target gene *her2* in control, MCT8 morphant and co-injection of MCT8 mRNA and MCT8 morpholino in zebrafish embryos at 25hpf. Lateral images of brain and spinal cord of analysed embryos are presented. In A,B,D scale bar represents 100μm. In C scale bar represents 25μm.

Supplementary Figure 8. Exogenous supply of MCT8 mRNA recovered the expression of genes affected by MCT8 MO to control levels in 25hpf zebrafish. WISH expression analysis of neural genes in control, MCT8 morphant and co-injection of MCT8 mRNA and MCT8 morpholino. (A) WISH expression analysis of neural progenitor marker *pax6a* in control. Lateral images of the hindbrain and spinal cord of analysed embryos are presented. (B) WISH expression analysis of neural progenitor factor *neurod6b*. Lateral (upper panel) and dorsal images (lower panel) of the brain of analysed embryos are presented. (C) WISH expression analysis of *retinoic orphan receptor ab* (*rorab*). Lateral images of the brain of analysed embryos are presented. (D) WISH analysis of expression of inhibitory neuron marker *gad1b*. Lateral images of the brain and spinal cord are presented. In all images scale bars represent 100μm.

Supplementary Figure 9. Exogenous supply of MCT8 mRNA recovered the expression of *flt4* to control levels in 25hpf zebrafish. WISH expression analysis of *flt4* in control, MCT8 morphant and co-injection of MCT8 mRNA and MCT8 morpholino. Lateral images of the head (first panel) and trunk (second panel) are shown. In all images scale bar represent 100μm.
